# Recovery from postpartum psychosis: a systematic review and metasynthesis of women’s and families’ experiences

**DOI:** 10.1007/s00737-020-01025-z

**Published:** 2020-02-04

**Authors:** R. Forde, S. Peters, A. Wittkowski

**Affiliations:** 1 Divison of Psychology and Mental Health, School of Health Sciences, Faculty of Biology, Medicine and Health, Manchester Academic Health Science Centre, The University of Manchester, 2nd Floor Zochonis Building, Brunswick Street, Manchester, M13 9PL UK; 2 Greater Manchester Mental Health NHS Foundation Trust, Manchester, UK

**Keywords:** Literature review, Qualitative research, Perinatal mental health, Psychotic disorder, *Childbirth*

## Abstract

Postpartum psychosis is a serious disorder that can result in adverse consequences for the mother and baby. It is important that we understand the experiences of women, to develop effective interventions during this critical period. The aim of this systematic review was to conduct a metasynthesis of qualitative research exploring women’s experiences of postpartum psychosis and factors involved in recovery from the perspective of women and family members. A comprehensive literature search of five databases was conducted and the findings were appraised and synthesised, following a thematic synthesis approach. Fifteen studies, capturing the views of 103 women and 42 family members, met the inclusion criteria. Four main themes incorporating 13 subthemes were identified following synthesis: (1) *Experiencing the unspeakable*, (2) *Loss and disruption*, (3) *Realigning old self and new self* and the integrative theme of (4) *Social context.* The findings offer new insight into the unique experience of postpartum psychosis and demonstrate that recovery does not follow a linear path. To improve clinical outcomes, a more integrative and individualised approach is needed which incorporates long-term psychological and psychosocial support, and considers the needs of the family. Further areas for staff training, service development and future research are highlighted.

## Introduction

Postpartum psychosis (PP) is one of the most severe mental health problems following childbirth, affecting 0.89 to 2.6 per 1000 women (Vanderkruik et al. 2017). It is characterised by a dramatic onset, and rapid deterioration, with symptoms including hallucinations, delusions, disorganised behaviour and depression (Di Florio et al. 2013; Heron et al. 2008; Sit et al. 2006). Continued poor maternal mental health is associated with increased risk of adverse outcomes including suicide and decreased mother-infant bonding (Wilkinson et al. 2017). Immediate referral to a secondary mental health service for assessment is therefore recommended (NICE 2018). Inpatient care, ideally within a Mother and Baby Unit (MBU; Gillham and Wittkowski 2015) and pharmacological intervention, is almost always required (NICE 2018; Jones and Smith 2009). Family members are reported to be integral during this process and should be involved in care and treatment planning (Engqvist and Nilsson 2014; Mohamied 2019).

With appropriate treatment, the most severe symptoms usually resolve within 2 to 12 weeks (Bergink et al. 2015). However, women remain at increased risk of subsequent postpartum and non-postpartum episodes, including depression and anxiety (Robertson et al. 2005; Nager et al. 2013). PP is a highly stressful life event which can have a detrimental impact on well-being and long-term functioning and result in feelings of guilt, loss, fear and shame (Burgerhout et al. 2017; Plunkett et al. 2016; Wittkowski et al. 2014). Psychological intervention and psychosocial support has been recommended (Doucet et al. 2011), yet very little is known about the psychological factors underpinning recovery or the types of intervention found to be effective.

Previous metasyntheses have explored individuals’ experiences of serious mental health problems and mental health services in the postnatal period (Ruffell et al. 2019; Smith et al. 2018; Plunkett et al. 2016; Megnin-Viggars et al. 2015; Wittkowski et al. 2014; Dolman et al. 2013). However, given the range of maternal mental health disorders included in those reviews, the applicability of these findings to women’s experiences of PP remains unknown. Thus, this systematic review and metasynthesis of published qualitative studies was conducted with the aims of (a) synthesizing the reported experiences of PP from the perspectives of women and family members and (b) identifying factors involved in recovery. The outcomes of which should be used to inform policy, practice and intervention development (Moore et al. 2015; Tong et al. 2012).

## Method

A metasynthesis was deemed most appropriate to systematically analyse and interpret multiple qualitative studies with the aim to develop new insight (Lachal et al. 2017). The review protocol was registered with PROSPERO on 19 December 2018 (Ref: CRD42018119145).

### Inclusion and exclusion criteria

All published empirical studies exploring women’s or family members’ experiences of PP and/or recovery using a qualitative methodology were included. Studies published in any language were eligible for inclusion, as were studies using mixed methods, provided qualitative data could be extracted.

Studies were excluded when they (1) explored ‘schizophrenia’ and other perinatal disorders, such as postnatal depression; (2) did not stipulate the diagnosis; (3) used a mixed sample in which it was not possible to differentiate the findings based on diagnosis; (4) explored only health professionals’ views; or (5) were unpublished research or grey literature.

### Search strategy

Five databases CINAHL, MEDLINE, EMBASE, PsychINFO and Web of Science were searched from inception to 3 April 2019. An updated search was completed on 20 August 2019 which revealed no additional eligible studies. Only keywords relating to the ‘phenomenon of interest’ (Cooke et al. 2012) were applied to ensure all relevant studies were captured (‘postpartum psychosis’ OR ‘puerperal psychosis’ OR ‘postnatal psychosis’ OR ‘psychosis after childbirth’) and no limits were applied. Additional studies were sought from the reference lists of included studies using forward and backward searching (Horsley et al. 2011).

### Study selection

**Fig. 1:**
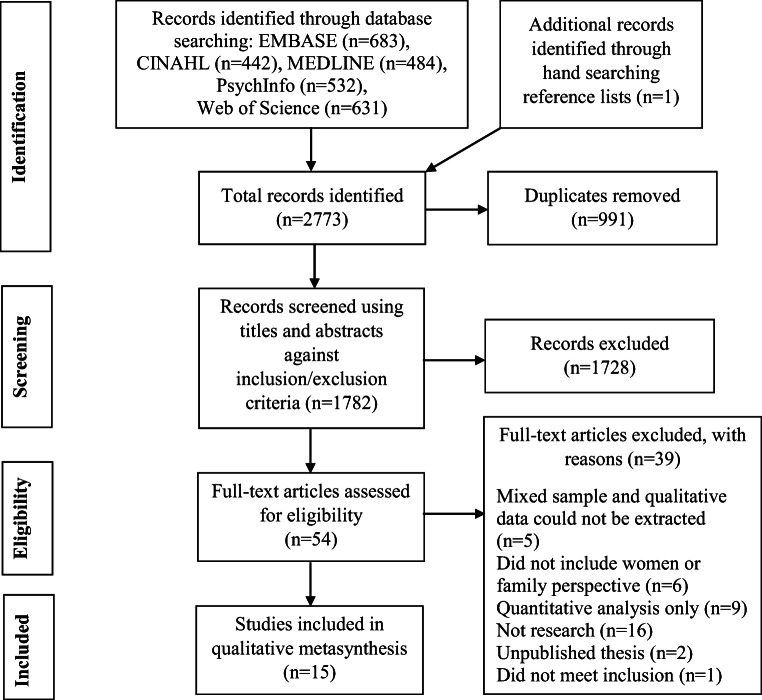
Summary flowchart of study selection and search outcomes

The search process, following PRISMA guidelines (Moher et al. 2009) yielded 1782 unique studies (see Fig. 1). The title, keywords and abstracts of all studies were assessed for eligibility against the inclusion/exclusion criteria; 1728 studies were subsequently excluded. A random 20% of studies (*n* = 353) were checked by an independent rater and a strong level of inter-rater reliability was obtained (96%, κ 0.88 (95% CI, 0.82–0.95) McHugh 2012). Of the remaining 54 studies, 39 were excluded when reviewed in full, yielding a final sample of 15.

### Critical appraisal

The methodological quality of the included studies was evaluated using the Critical Appraisal Skills Programme (CASP 2018) checklist. The CASP comprises ten questions across three broad domains, relating to the validity, design and utility of the results. The CASP was used to determine rigour and trustworthiness in the outcomes (Hannes 2011).

### Data analysis

All included studies were extracted electronically onto NVIVO 12 (2018) data management software and analysed using Thomas and Harden’s (2008) thematic synthesis (see Table 1). This approach enabled the researchers to integrate multiple qualitative findings and identify recurring themes, following three key overlapping stages (Noyes et al. 2018; Ring et al. 2011). Distinctions were made across the different samples when analysing the data and constant comparisons were made across and within the women’s and family members’ accounts to ensure similarities and differences were captured. To test the credibility and applicability of the findings, a peer and member-checking exercise was completed during theme development stage (Lincoln and Guba 1985; Creswell and Miller 2000).

**Table 1:** Thematic synthesis process

Stages of thematic synthesis	
1	Free line-by-line coding completed inductively across all data contained within the ‘findings/results’ sections of each study, including participant quotes, author narrative and interpretation. The women’s accounts were coded first, followed by those of family members. New concepts were created according to the meaning and content of each sentence.
2	These codes were then organized into related areas and descriptive themes were developed, using an iterative process which involved the refinement of codes, checking for consistency of interpretation.
3	Higher level analytical themes were generated with the aim of producing new interpretations and key messages as guided by the research aims. This was continuously reviewed until a final set of analytical themes that captured the key meanings across the women’s and family members’ accounts were agreed upon.

## Results

### Study characteristics

The qualitative synthesis comprised 15 studies reporting the experiences of 103 women and 42 family members, of which 32 were partners (see Table 2). All studies were published between 2003 and 2018. Ten studies were conducted in the UK, two in Sweden, two in the USA and Canada and one used online data (location unknown). A diverse sample was obtained, in which women who participated in the studies were reported to be at different stages of their recovery, ranging from 2 months postpartum to 32 years. No women were reported to be actively psychotic during the interview and this was frequently cited as an exclusion criteria to ensure informed consent could be obtained. Engqvist et al. (2011) used online narratives as a data source and therefore the duration since onset could not be determined. The duration since onset reported by family members was also broad, capturing experiences up to 19 years postpartum. One study (Boddy et al. 2016) specifically sought individuals’ experiences during the acute phase of psychosis and this was obtained from the perspective of fathers only.

**Table 2:** Summary of study characteristics

Study: authors, year, location		Study aim	Sample description	Method	Data collection	Method of analysis	Overview of identified themes
Studies investigating women’s perspectives only (in reverse chronological order)							
1	Stockley (2018) UK	Examine women’s experiences during the onset and early days of PP	Seven women—all identified as having PP Aged Time since onset: 2–22 years	Advertised on Action on Postpartum Psychosis (APP)	Face-to-face semi-structured interviews (20–49 min)	IPA (Smith et al. 2009)	1. What’s happening?: sleep deprivation and anxieties and losing touch with reality 2. ‘Lack of recognition of the seriousness’: keeping up appearances and misinterpretation 3. Breast is best?: difficulties and anxieties related to feeding 4. ‘Trauma’: both prior to and during birth
2	Roberts et al. (2018) UK	How the EastEnders PP storyline and increase in public awareness were received by women recovered from PP	Nine women—fully recovered from PP Time since onset: at least 12 months	Advertised on APP	Face-to-face (*n* = 1) and telephone (*n* = 8) semi-structured interviews (43–97 min)	TA (Braun and Clarke 2006)	1. Public education: PP as a hidden illness, importance of improving understanding 2. Stigma: viewed as deep rooted 3. Disclosure: difficulty sharing experience of PP, storyline facilitated disclosure 4. Reassurance: they were not alone 5. Family relationships: largely negative impact on family, including distress and trauma—storyline as a vehicle to understanding PP from their perspective
3	Plunkett et al. (2017) UK	Explore the role of the baby in recovery from PP	Twelve mothers experienced PP Aged 23–56 years Time since onset: 2 months–26 years	Advertised on website forums (n = 9) and MBU (n = 3)	Face-to-face (*n* = 5) and telephone (*n* = 7) semi-structured interviews (22–52 min)	TA (Braun and Clarke 2006)	1. The baby has a role in recovery: motivated mothers to get better 2. The baby is a barrier: responsibility of caring created emotional distress; societal expectations delayed help-seeking 3. Baby facilitates recovery: physical contact reduced distress and increased maternal self-efficacy
4	Glover et al. (2014) UK	Gain further insight into women’s experiences of PP and the context in which they make sense of it	Seven women Aged 25–45 years All diagnosed with PP in the last 10 years	Specialist psychiatry services for mothers and babies	Semi-structured interviews Location not specified (approximately 1 h)	TA (Braun and Clarke 2006)	1. The path to PP: negative perception of birth experience and sense of detachment 2. Unspeakable thoughts and unacceptable self: unbearable thought content 3. Snap out of it: women felt dismissed and judged, exacerbated distress 4. Perceived causes: PP unavoidable vs ‘snap out of it’: an unpreventable illness
5	McGrath et al. (2013) UK	Develop a theoretical understanding of recovery from psychosis following childbirth	Twelve women Aged 26–45 years Time since onset: 4 months–23 years	MBU (*n* = 2) and online advertisements (*n* = 10)	Face-to-face (*n* = 11) and telephone (*n* = 1) semi-structured interviews (37–110 min)	Constructivist grounded theory (Charmaz 2006)	Recovery conceptualised as a parallel process 1. The process of recovery: from ‘immobilisation’ (unable to make use of active strategies) to recognising changes and accepting loss self-efficacy and hope 2. Evolving an understanding: dynamic process, recognise mismatch in expectations 3. Strategies for recovery: initially felt powerless and tried to conceal symptoms 4. Sociocultural context
6	Posmontier and Fisher (2013) USA	Understand the experience of PP in an Orthodox Jewish woman	One Orthodox Jewish woman Time since onset of PP: 2 years	Not specified	Unstructured Telephone interview (1.5 h)	Structural analysis (Gee 1991)	First day: birth and Shavuos (Jewish holiday), Second day: the symptoms begin—a sleepless night and a sense of dread Third day: coming home Fourth day: Shabbos psychosis—more agitated, directive and loss of trust The aftermath: making sense of experience
7	Heron et al. (2012) UK	Explore women’s experiences of the process of recovery and their beliefs about the services needed	Five women—regarded themselves as fully recovered Time since onset: 3–20 years	Expressed interest in conducting research via APP	Service user led face-to-face semi-structured interviews (19–45 min)	Grounded analytic induction approach (Silverman 2006)	1. Unmet expectations: psychological enormity and sense of loss 2. Ruminating and rationalising: a need to integrate periods of lost time and confusion 3. Social recovery: building networks 4. Medical support: considered vital 5. Information needs: should be tailored 6. Family functioning: support seen as pivotal 7. Giving recovery time: not always linear
8	Engqvist et al. (2011) Anonymous data	Gain a deeper insight into women’s experiences of PP, as described in narratives published on the internet	Ten personal narratives taken from the Internet Of 28, 10 met the DSM-IV criteria for PP	Internet search for narratives written by women who describe PP	Internet as a data source (306–4140 words)	Content analysis (Krippendorff 2004)	1. Unfulfilled dreams: shattered expectations 2. Enveloped by darkness: women felt an overwhelming fear, were in an unreal world and experienced disorganised thinking 3. Disabling symptoms: lonely, suspicious, loss of sleep and self-destructive behaviours 4. Being abandoned: all felt distrusting and detached from others
9	Robertson and Lyons (2003) UK	Explore women’s experiences of PP and gain understanding into living through and past the illness	Ten women Aged 28–44 years Diagnosed with PP in the last 10 years	Subsample from a previous genetic study	Face-to-face semi-structured interviews (40–90 min)	Grounded theory principles (Glaser and Strauss 1967)	1. A separate form of mental illness 2. Loss: women felt powerless 3. Relationships and social rules: all time and effort focussed on the baby Higher order concepts: 1. Living with emotions: guilt and loss 2. Regaining and changing self: feeling like themselves again, marker for recovery
Studies including family members’ perspectives (in reverse chronological order**)**							
10	Holford et al. (2018) UK	Consider the lived experiences of partners of women who have had PP and the impact that it has had on their lives	Eight male partners Aged 30–49 years All partners experienced PP in the last 10 years and longer than 6 months since onset.	Advertised on APP	Face-to-face (*n* = 1), telephone (*n* = 4) and skype (*n* = 3) semi-structured interviews	IPA (Smith et al. 2009)	1. Loss: unmet expectations, resulting in grief and a sense of abandonment 2. Powerlessness: general lack of control 3. United vs. individual coping 4. Hypothesising and hindsight: benefit of reflection to make sense 5. Barriers to accessing care and unmet needs: poor understanding and empathy 6. Managing multiple roles 7. Positive changes from PP
11	Boddy et al. (2016) UK	Explore fathers’ experiences during their partners’ MBU admission for PP	Seven men during partners’ admission to an MBU Aged 23–42 years	Two MBUs whilst partners were receiving inpatient care	Face-to-face semi-structured interviews (40–84 min)	IPA (Smith et al. 2009)	1. ‘What the f*** is going on?’ - PP as an unexpected arrival - Not feeling heard 2. ‘Time to figure out how your family works’: - Holding the fort - Loss and reconnection - Adjustment to family life
12	Wyatt et al. (2015) UK	Explore how women and their significant others make sense of their experience of PP and their relationships	Seven women with PP and significant other Women aged 28–33 years, significant other: 29–39 years Time since onset: 5 months–4 years	Dyads identified through a perinatal service (*n* = 1) and social media (*n* = 6)	Dyad face-to-face semi-structured interviews (60–85 min)	IPA (Smith et al. 2009)	1. ‘She wasn’t herself’: threatened relationships through loss of ‘normal’ self 2. Invalidation and isolation: distress felt unrecognised or minimised 3. ‘The worst life can throw at us’: shared perceptions of trust and respect after PP 4. A double-edged sword: understanding the influence of relationships on PP
13	Engqvist and Nilsson (2014) Sweden	Explore the recovery process of PP and conclusion of hospital care from the perspective of women and next of kin	Seven women with PP and six next of kin Women aged 44–62 years, time since onset: 7–32 years Next of kin aged 39–72, time since onset: 6 months–19 years	Contact initiated via an interview at a local radio station and snowball sampling	Face-to-face semi-structured interviews (45–90 min)	Content analysis (Graneheim and Lundman 2004)	1. The recovery: ‘Return to life’ - The turning point: from being trapped - Own recovery: returning strength - Social recovery: ability to socialise 2. Supporting circumstances: to regain their health—relatives’ and friends’ support, professional support and support through medication 3. Light in the tunnel of darkness: an internal decision to get well
14	Engqvist and Nilsson (2013a) Sweden	Explore accounts of the first days and early signs of PP from the perspectives of women and next of kin	Seven women with PP and six next of kin Women aged 39–60 years, time since onset: 7–32 years Next of kin age unknown, time since onset: 5 months–19 years	Contact initiated via an interview at a local radio station, and snowball sampling	Face-to-face semi-structured interviews (45–90 min)	Content analysis (Graneheim and Lundman 2004)	Main theme: shades of black with a ray of light—darkness, despair and suffering, next of kin viewed PP as incomprehensible 1. Loss of sleep: exhaustion 2. Being in an unreal world 3. From wanting the baby to not wanting the baby: gave rise to guilt and shame 4. Infanticidal ideation: did not trust self 5. Suicidal ideation: complete darkness
15	Doucet et al. (2012) Canada and USA	Explore the support needs, preferences, accessibility to resources, and barriers to support	Nine women and eight fathers/partners Mean age of women: 34.7 years, mean age of men: 36.3 years Time since onset: up to 10 years	Purposive sampling—community and hospital agencies	Face-to-face (*n* = 8) and telephone (*n* = 9) semi-structured interviews (45–120 min)	TA (Braun and Clarke 2006)	1. Support needs: relating to generic parenting needs and serious mental illness 2. Support preferences: informational from professionals and emotional support from informal networks 3. Availability and accessibility: nonexistence of specialist support, majority of support provided by family 4. Barriers to support: health service barriers and lack of knowledge about PP

Using the CASP checklist, no major methodological issues were identified and all 15 studies were rated to have high methodological quality and low methodological bias (see Table 3).

**Table 3:** Outcomes of the CASP checklist for the 15 qualitative studies included in the metasynthesis

		1	2	3	4	5	6	7	8	9	10	11	12	13	14	15
1.	Was there a clear statement of the aims of the research?	Yes	Yes	Yes	Yes	Yes	Yes	Yes	Yes	Yes	Yes	Yes	Yes	Yes	Yes	Yes
2.	Is a qualitative methodology appropriate?	Yes	Yes	Yes	Yes	Yes	Yes	Yes	Yes	Yes	Yes	Yes	Yes	Yes	Yes	Yes
3.	Was the research design appropriate to address the aims of the research?	Yes	Yes	Yes	Yes	Yes	Yes	Yes	Yes	Yes	Yes	Yes	Yes	Yes	Yes	Yes
4.	Was the recruitment strategy appropriate to the aims of the research?	Yes	Yes	Yes	Yes	Yes	PA*	Yes	Yes	Yes	Yes	Yes	Yes	Yes	Yes	PA*
5.	Was the data collected in a way that addressed the research issue?	Yes	Yes	Yes	PA*	Yes	Yes	Yes	Yes	Yes	Yes	Yes	Yes	Yes	Yes	PA*
6.	Has the relationship between researcher and participants been adequately considered?	Yes	Yes	PA*	No	Yes	Yes	PA*	Yes	Yes	Yes	Yes	Yes	Yes	No	No
7.	Have ethical issues been taken into consideration?	Yes	Yes	Yes	Yes	Yes	PA*	Yes	Yes	PA*	Yes	Yes	Yes	Yes	Yes	Yes
8.	Was the data analysis sufficiently rigorous?	Yes	Yes	Yes	Yes	Yes	Yes	Yes	Yes	Yes	Yes	Yes	Yes	Yes	Yes	Yes
9.	Is there a clear statement of findings?	Yes	Yes	Yes	Yes	Yes	Yes	PA*	Yes	Yes	Yes	Yes	Yes	Yes	Yes	Yes
10.	How valuable is the research?	Yes	Yes	Yes	Yes	Yes	Yes	Yes	Yes	Yes	Yes	Yes	Yes	Yes	Yes	Yes

*PA = partially agree

### Synthesis

**Fig. 2:**
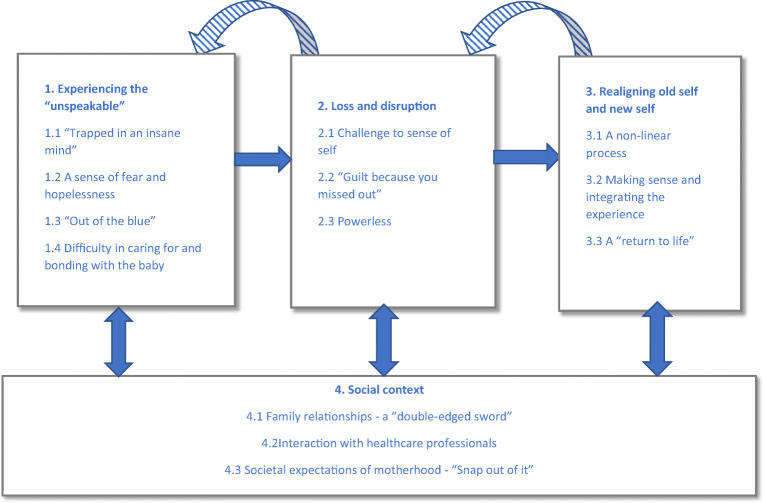
A conceptual model of women’s and family members’ experiences of postpartum psychosis and the factors involved in recovery

Four main themes and 13 subthemes were conceptualised in the metasynthesis, representing the reported experience of PP and factors involved in recovery from the perspective of women and family. A conceptual model was developed (see Fig. 2) to provide a visual representation and to illustrate the relationships between themes. This captured themes from both women and family members, due to the similarities found in some of their experiences. The model demonstrates women’s non-linear progression towards recovery, in which they often moved back and forth between phases and this process was integrated within the wider social context. Illustrative quotes are provided below each theme with additional data presented in Table 4.

**Table 4:** Thematic structure with Illustrative quotes

Theme	Illustrative quotes
Theme 1: experiencing the ‘unspeakable’	
1.1: ‘Trapped in an insane mind’	‘For in my paranoia I was certain that my husband (who really is one of the world’s greatest men and husbands) was out to get me. I thought he wanted to divorce me and take our child. I thought he was probably sabotaging our efforts to get help. This man, who I trust more than anyone in the world, I felt I could not trust’ (Woman p.383; Engqvist et al. 2011). ‘It feels like it happened to another person, it’s hard for me to say how I felt. I felt like, throughout most of it… like I was watching someone else do it’ (Woman p.155; Stockley 2018). ‘She was ranting and raving and my friends.. .. both looked at me and I was like, what the fuck is going on’ (Partner p.402; Boddy et al. 2016).
1.2: A sense of fear and hopelessness	‘I just felt very lonely, very homesick. I felt like no one was really paying attention to me. I was just thrown into this place... left all alone not knowing how to cope or what to do or anything’ (Woman p.171; Posmontier and Fisher 2013).
1.3: ‘Out of the blue’	‘If I’d known about it [postpartum psychosis] I’d probably have, I’m not saying it would have stopped it, but it probably would have helped to understand a little thinking ‘this is what I’m having’, because obviously if you have never heard of anything and you go off your rocker, you think my God, what is wrong?’ (Woman p.80; Roberts et al. 2018).
1.4: Difficulty in caring for and bonding with the baby	‘I wanted to go to sleep so I would turn over, and I′d be like, I do not want to be with him [baby] now’ (Woman p.1103; Plunkett et al. 2017). ‘It was a horrible feeling. I felt like her milk was dirty and I used to forget. My memory went, I would forget everything… I do not know how many bottles I threw away ‘cause I was so paranoid of making the baby sick. I was paranoid about everything’. (Woman p.156; Stockley 2018) ‘I found for a long time it was at least a two-person job to manage things. Because one person had to take care of the baby, and usually I had to take care of my wife’ (Partner p.241; Doucet et al. 2012). ‘This was something completely different. I thought she handled our son recklessly. She held him almost as if he were a doll’ (Partner p.85; Engqvist and Nilsson 2013a).
Theme 2: loss and disruption	
2.1: Challenge to sense of self	‘Your whole being, how you see yourself, the kind of person you are, and your whole sense of identity is completely devastated really, because you will have behaved in ways that shock you completely. I’m not a, you know, successful person anymore that I thought I was, I’m now a mental health patient’ (Woman p.158; Heron et al. 2012). ‘My daughter became a complete stranger. She was totally deflated... It seemed as if she was totally lost in the world. You just do not know what to do. It was difficult to get through to her and in [a] way she was inside a fog all by herself’ (Parent p.85; Engqvist and Nilsson 2013a). ‘I worried about interaction. I worried that I’d never get better from the depression because it was very cyclical. I really, really worried about the impact on [my baby] because I’d read about mums who have mental health problems affecting their kids, I worried about the genetic side of it. What did not I worry about? I worried for my marriage, all sorts of stuff like that really. I worried for the future really—being ill, thinking am I always, at that point because, I did think that I was never going to get better, so that was my main sort of fear’ (Woman p.158; Heron et al. 2012).
2.2: ‘Guilt because you missed out’	‘All these feelings of guilt because you missed out, or not being there, for your new born baby, and guilty, because you left your husband to deal with it all when it’s the first time for him as well’ (Woman p.157; Heron et al. 2012). ‘You would think that the first time after having a baby you go round obviously showing her off to everybody, and we could not do that purely because [the mother] wasn’t there’ (Partner p.3; Holford et al. 2018).
2.3: Powerless	‘My family said I wasn’t well and that I needed to take tablets but they never explained to me why or what that illness was… No leaflets, no information, and well the attitude was quite, you know, take the tablets and do not say nothing’ (Woman p.160; Heron et al. 2012). ‘For the first five days, all I got was, ‘you are not married, your son’s not registered, you have got no right to know where they are or what’s going on’ (Partner p.403; Boddy et al. 2016).
Theme 3: realigning old self and new self	
3.1: A non-linear process	‘I thought ‘I’m out of the woods’ you know…‘yeah it’s all going to be fine’, and then actually the depression afterwards, the deep, deep depression afterwards, was just such a blow, such a double whammy’ (Woman p.162; Heron et al. 2012).
3.2: Making sense and integrating the experience	‘At least I wanted someone to talk to, which I got when my son was about one year old. And I concluded this contact now, barely three weeks ago. So, it has taken 18 years to deal with this backpack of mine...!’ (Woman p.12; Engqvist and Nilsson 2014). ‘I’m now a specialist in postpartum psychosis medication involved, because I am one of those people who will research in terms of Google and APP networks and whatnot. I guess that had been my idea of finding out what is actually going on’ (Partner p.5; Holford et al. 2018). ‘I actually saw a bloke who was a trainee clinical psychologist, but he was brilliant. He just gave me time to cry and be upset and talk about all these worries. And with CBT, which is a sort of talking treatment, he helped me to find different ways into that negative cycle of worries’ (Woman p.159; Heron et al. 2012).
3.3: A ‘return to life’	‘I just feel very proud of myself’ (Woman p.174; Posmontier and Fisher 2013). ‘Slowly, my wife was becoming her old self once again’ (Partner p.12; Engqvist and Nilsson 2014). ‘I can stand back and look at myself in the past when I was ill and that is not me. I went from being a confident woman to, I cannot think of a word, well erm pathetic that’s what I was, now I’ve regained my confidence and I feel better than I did before because I’ve been through this and come out stronger. Nothing as bad can ever happen to me again’ (Woman p.424, Robertson and Lyons 2003).
Theme 4: social context	
4.1: Family relationships—a ‘double-edged sword’	‘If they [family] understand then they will just step up more’ (Woman p.239; Doucet et al. 2012). ‘I needed advice on how to handle the illness and what to say. Also, information on the early signs of relapse to watch for and if it was to the point that I needed to get help’ (Partner p.241; Doucet et al. 2012). ‘It’s as if I did not have enough love to go round and so I gave it all to my son’ (Woman p.421; Robertson and Lyons 2003).
4.2: Interaction with healthcare professionals	‘I was on a very high dose of Olanzapine and it just knocks you out and makes you into a complete zombie. The psychiatrist was a young guy not understanding that we had needs as a family. My husband really needed me to be awake enough to get my baby dressed and you know, do that kind of stuff’ (Woman p.159; Heron et al. 2012). ‘Those places [MBU] are a lifesaver, because if you are not in that specialism no one else can really understand’ (Partner p.7; Holford et al. 2018). ‘That would be the number one reason for me not telling anyone. Cos I was utterly convinced if I told the doctor I am thinking of throwing my baby out of the window … they are going to think ‘Oh my god that poor baby.’ And you know you hear that from the paper that they were taken away and that’s it. I did not tell a soul’ (Woman p.1103; Plunkett et al. 2016)
4.3: Societal expectations of motherhood—‘Snap out of it’	‘I feel that people often think it’s postnatal depression, and they do not understand it at all. It’s taken me 20 years of really banging my head against the wall trying to make people aware of it’ (Woman p.77; Roberts et al. 2018). ‘There was never much discussion, even from family, but certainly not from any of the medical services, as to how I was, what concerns did I have. I was very much left to feel that you have got to cope’ (Partner p.6; Holford et al. 2018).

### Theme 1: experiencing the ‘unspeakable’

During the initial stages of PP, women reported ‘shock’ (Women p.157; Heron et al. 2012) and their experiences were conceptualised as ‘unspeakable’ (Author p.260; Glover et al. 2014).

#### ‘Trapped in an insane mind’

Women reported a range of distressing thoughts, which often related to their newborn baby ‘Did I kill my baby?’ (Woman p.240; Doucet et al. 2012). Women reported experiencing racing thoughts and unusual perceptions; they worried that their baby would be taken away from them and felt ‘trapped in an insane mind’ (Woman p.380; Engqvist et al. 2011). These fears led women to mistrust people close to them and attempts to conceal their symptoms contributed to delays in families seeking help. Partners reflected that they missed earlier sign and referred to a key, sometimes ‘traumatic’ moment (Partner p.402; Boddy et al. 2016), where it became clear they needed help.

#### A sense of fear and hopelessness

Many women described their experience as traumatic, frightening and overwhelming ‘I never knew this kind of fear or darkness existed’ (Woman p.381; Engqvist et al. 2011). This sense of fear was often exacerbated by women’s hospital admission and associated separation and isolation from their family ‘I thought I was on my way to hell on earth’ (Woman p.381; Engqvist et al. 2011). Women described feeling out of control and worried they were ‘never going to get better’ (Woman p.158; Heron et al. 2012). A similar sense of hopelessness was reported by the family:

When everything is just dark, and every day is a hell to get through, then it’s tough. It’s terrible. The days are hopeless (Father p.87; Engqvist and Nilsson 2013a).

#### ‘Out of the blue’

Women and family members consistently reflected on the sudden and severe escalation of PP. They reported having minimal to no information prenatally, which added to their sense of being unprepared*.* As women and family members tried to make sense of why the woman had developed PP, many attributed the diagnosis to a biological cause involving a hormonal imbalance and factors related to pregnancy, such as sleep disturbance or a traumatic birth experience. These birth-specific attributions helped women and families to view PP as a disorder specific to childbirth that can happen ‘out of the blue’ to anyone (Woman p.78; Roberts et al. 2018), thereby externalising the cause and reducing self-blame.

#### Difficulty in caring for and bonding with the baby

During the early phase of PP, many women experienced increased anxiety, reduced confidence and reported delays bonding with their babies. Women described meeting their babies’ physical needs, but with a sense of detachment:

I fed him, bathed him, changed him and was able to make up his feeds, but I had no real affection for him (Woman p.383; Engqvist et al. 2011).

Women reflected on how difficult they found caring for their baby, when they were extremely exhausted themselves and sedated from medication or fearful of accidentally causing harm. As a result, women often relied on family members and nursing staff to provide practical care for their baby. For some partners, providing the level of support needed for both their baby and partner became extremely challenging:

I honestly don’t think I could have survived much longer (Partner p.4; Holford et al. 2018).

MBU admission was often described as a relief for family members because they believed both the woman and baby were safe and being cared for.

### Theme 2: loss and disruption

Women and family reported a sense of loss and disruption that permeated across multiple areas of their lives and remained with women beyond the remission of their acute psychotic symptoms.

#### Challenge to sense of self

The experiences associated with the acute stage of PP challenged women’s sense of self and personal identity. Women and family members described the woman’s presentation as being significantly out of character and resulting in feelings of loss, abandonment and fear for the future:

I did not recognise her at all… it just wasn’t her… I was just terrified….that I had lost her (Partner p.4; Holford et al. 2018).

Women’s experiences of loss remained with them beyond recovery and women believed they had ‘undergone a change that was likely permanent’ and this change required acceptance (Woman p.340; McGrath et al. 2013).

#### ‘Guilt because you missed out’

As time progressed, women reflected on multiple losses. In particular, they believed they had missed out on the first few months of their child’s life and worried about the impact of this on their child’s development. Many women and partners described guilt and disappointment resulting from unfulfilled expectations of parenthood. Some reported a sense of shame for the initial thoughts they had about their baby: ‘I was appalled that I could have such an awful thought’ (Woman p.382; Engqvist et al. 2011). Some parents decided against having further children due to the increased risk of a subsequent episode of PP. A decision that was often associated with additional feelings of sadness and loss:

I loved being pregnant and I loved carrying a child… not to do that is heartbreaking (Woman p.420; Robertson and Lyons 2003).

#### Powerless

A sense of powerlessness was evident across the accounts of women and family members. In the early stages, women were reliant on family, friends and healthcare staff for information and support which often left them feeling ‘helpless and disempowered’ (Author p.160; Heron et al. 2012). Family members regretted things they ‘should have’ done and felt as though they had ‘let the mother down’ (Partner p.6; Holford et al. 2018), particularly in relation to their involvement in the women’s hospital admission. Family members described being ignored and expressed frustration at being denied information due to patient confidentiality.

### Theme 3: realigning old self and new self

Recovery was described as a lengthy process which involved women making sense of and integrating their experiences, as well as identifying personal strength and adjusting to a new view of self, in the context of being a parent.

#### A non-linear process

The ‘very, very long journey’ (Woman p.174; Posmontier and Fisher 2013) to recovery was described as following a ‘progressive but non-linear trajectory’ (Author p.163; Heron et al. 2012). During this process women reported anxiety, uncertainty about the future and periods of low mood and depression, which occasionally necessitated a hospital readmission. Recovery was conceptualised as an ‘active process’ (Author p.5; McGrath et al. 2013) in which women became motivated to develop their understanding and utilised strategies to reduce the risk of further recurrence.

#### Making sense and integrating the experience

The ability for women to openly disclose their experience was regarded as important for recovery. In order to make sense of why they had experienced PP, many attempted to organise events in time and sought information about the disorder. Developing their understanding helped to alleviate self-blame. Communicating with others with similar experiences and sharing their story was important to foster feelings of hope and reduce feelings of isolation and self-blame.

Women wanted counselling or psychotherapy for themselves and also the wider family to help express and come to terms with their experience. However, barriers to accessing support were reported, including lack of provision of talking therapies and different needs within the family sometimes hindered women’s ability to talk through and integrate their experiences:

He could not understand at times why I just could not pull myself together which annoyed me even more (Woman p.421; Robertson and Lyons 2003).

#### A ‘return to life’

Women spoke of turning points in which they started to feel more hopeful about the future and made a decision to ‘return to life’ (Woman p.10; Engqvist and Nilsson 2014). Positive experiences, such as connecting with other women and bonding with the baby helped to enhance women’s self-efficacy. Experiences such as these enabled women to feel like ‘themselves again’ (Author p.424; Robertson and Lyons 2003) and provided hope for the family that recovery was ‘within reach’ (Author p.13; Engqvist and Nilsson 2014).

Whilst describing the experience of PP as traumatic, some positive aspects were identified. Women reported feeling more confident or stronger in themselves:

Before I just thought well, I’ll coast along until whenever and life’s not that bad but now I’m not really scared of anything (Woman p.424; Robertson and Lyons 2003).

Women valued those close to them, reported greater empathy and often felt motivated to help others and ‘give something back’ (Woman p.424; Robertson and Lyons 2003). Family members similarly reported improved relationships, increased empathy, openness and understanding, both in relation to their family member and more generally towards people experiencing mental health difficulties.

### Theme 4: social context

Social context was conceptualised as an integrative theme that both influenced and was influenced by the women’s experience of recovery from PP.

#### Family relationships—a ‘double-edged sword’

Throughout the process of recovery, family were concurrently viewed as a ‘source of immense support and a source of worry’ (Woman p.161; Heron et al. 2012). Women valued the support provided by family but simultaneously worried about their families’ well-being. In the early stages, women expressed guilt for burdening their family and sometimes expressed strain within their relationships. Women reported prioritising their relationship with their child, but sometimes felt this was to the detriment of their other relationships. Partners reflected on their own stress throughout this experience, but felt unable to seek help:

I was an emotional wreck but felt I had to gather myself together for my wife... I could not have any issues, someone had to be strong (Partner p.241; Doucet et al. 2012).

Psychological support for families was needed to help develop family members’ understanding of PP, enhance their coping with the additional stress and enable them to know how to respond and cope with fear around relapse:

If my husband had a support group for new fathers to deal with a psychotic wife, it would have changed everything. He would have been far more compassionate had he known about my illness (Woman p.241; Doucet et al. 2012).

#### Interaction with healthcare professionals

Interactions with mental health services were perceived by women and family members as both a facilitator and barrier to recovery. In one study, women reported a sense of pressure to appear to be coping; this was associated with a fear of having their baby removed and acted as a barrier to seeking professional input (Plunkett et al. 2016). Some women and family members reported difficulties accessing support, felt there was a divide between hospital and community services and believed there had been little consideration of their needs as a family. Women and family members occasionally felt judged by health care professionals ‘You do the smallest thing and, oh no, you’re doing it wrong’ (Partner p.405; Boddy et al. 2016).

Some reported more positive experiences and felt ‘cared for’ (Woman p.384; Engqvist et al. 2011). Positive experiences improved women’s confidence, promoted their relationship with their baby and provided reassurance to the family, many of whom valued the expertise and support provided by healthcare professionals.

#### Societal expectations of motherhood—‘snap out of it’

Women and family members both highlighted the public’s limited understanding of this ‘hidden illness’ (Woman p.77; Roberts et al. 2018) which was frequently confused with postnatal depression. The lack of awareness by professionals and peers prevented women from seeking and gaining support and left women feeling as though their symptoms were minimised or dismissed. Some women experienced an expectation from their personal network to ‘snap out of it’ (Woman p.261; Glover et al. 2014) which exacerbated a sense of internal shame and hindered seeking help:

It’s a double whammy. Not only the stigma of being mentally ill, you have got the stigma of being a mentally ill mother, a bad mum (Woman p.78; Roberts et al. 2018).

## Discussion

This is the first review to synthesise the qualitative research on the experience of PP and the factors involved in recovery. The findings provide further evidence as to the extreme and distinct nature of PP (Di Florio et al. 2013) in which there are unique aspects to recovery that should be recognised and inform care provision. The findings of this review reveal that recovery can be a lengthy and non-linear process that continues beyond acute symptom remission and is influenced by the wider social context. Furthermore, the review reveals similarities in the experiences of women and family members, including a reported sense of shock, fear for the future, hopelessness and difficulties in coping and seeking help.

During the early phase, the distressing nature of PP left women and family members struggling to identify what was wrong and unable to access information. Perceived societal stigma and fear of negative repercussions delayed help-seeking behaviour; this may be associated with fear of losing their baby; however, further investigation is required. In their review looking at mental health problems in the postnatal period, Megnin-Viggars et al. (2015) similarly found that women often reached crisis point before seeking help. This finding is important, given the increasing rates of suicide amongst postpartum women experiencing psychosis (Lysell et al. 2018) and the potential negative consequence for the mother-baby relationship (Alhusen et al. 2013). The reported delays in help-seeking behaviour provide further support for NICE (2018) guidelines which highlight the importance of healthcare professional vigilance to possible symptoms of PP and provision of prompt assessment and intervention.

The findings reveal contact with healthcare services is inconsistent and can even hinder accessing support, suggesting that NICE guidelines are not being consistently met. This may reflect differences in service provision, including insufficient availability of specialist MBUs which can result in delays receiving appropriate help (Hill et al. 2019; Jones and Smith 2009). It was also found that the needs as a family were often not adequately addressed, echoing work in the broader area of maternal mental health (Megnin-Viggars et al. 2015; Dolman et al. 2013) and providing further evidence to provide healthcare staff with specialised training on PP to enhance their skills and confidence (Dolman et al. 2013).

Perceived loss has featured in previous reviews relating to severe maternal mental health disorders (Smith et al. 2018; Wittkowski et al. 2014; Dolman et al. 2013). However, for women and families experiencing PP, the sense of loss was pervasive and reported across multiple domains of women’s lives. Women feared losing their child and reported loss of time, control, freedom and perceived loss of subsequent children. These losses all disrupted the women’s adjustment to motherhood. Time and support were needed to reflect on these losses and to explore their new identity. Dolman et al. (2013) similarly found that women experiencing severe mental health disorders during motherhood need to integrate their ‘dual identity’ of being a ‘woman with a mental illness’ and a ‘mother’ for successful transition to occur. Furthermore, across the studies, women and partners reported a sense of loss in relation to their decision-making about future pregnancies. Although NICE (2018) guidelines recommend providing preconception advice for those at risk of mental health problems, the findings of this review suggest this support may need to be more proactive and ensure the emotional impact of this decision is incorporated.

The centrality of family support was clearly communicated throughout participants’ narratives and confirms previous reports that family plays a ‘key role’ (p.3; Plunkett et al. 2016). Differences were noted in the family members’ ability to cope and provide the level of support needed to manage at home. Improved understanding of postnatal mental health problems has been found to influence the relationship and can result in greater resilience and confidence by the partner (Ruffell et al. 2019). The importance of involving the family by providing information and fostering feelings of security and hope has also been reported by professionals working with women experiencing PP (Engqvist and Nilsson 2013b; Engqvist et al. 2007). However, as family perceived a need to be ‘strong’, this sometimes acted as a barrier to seeking additional external help for themselves.

### Clinical implications

In order to improve outcomes and facilitate women’s recovery, recommendations based on women’s and family members’ reported experiences are provided in Table 5. Additional recommendations are suggested for healthcare professionals. However, given that the included studies did not report on the views of healthcare providers, these recommendations should be treated with some caution.

**Table 5:** Suggested clinical implications and recommendations

Women	• Accurate information should be provided, alongside access to peer support to help normalise women’s experiences. • Women could be offered psychological and psychosocial input during the latter stages of recovery not just the early stages. • Longer term psychological needs should be considered, incorporating the reported feelings of guilt, loss and difficulties transitioning to their new perceived role. • Practical considerations are required to enable access to psychological support, this should be flexible and consider childcare provision. • Women/partners should be offered more proactive support when reaching decisions about future pregnancies.
Family	• The needs of the family should be considered and incorporated into any assessment and intervention plan as much as possible. • Family members’ wellbeing should be monitored and they should be signposted to appropriate support and peer networks as required. • Family members should be informed of reputable sources to obtain accurate information. • Families may benefit from a therapeutic space in which they can openly explore and seek to resolve any difficulties within their relationships.
Healthcare professionals	• Specialist training and support may help to develop healthcare professionals’ confidence and competence meeting the complex needs of women experiencing PP and their family. • It is important that professionals maintain a compassionate and non-judgmental stance; in order to develop a therapeutic relationship which promotes optimism and hope for the future. • Healthcare professionals should pay particular attention to women who do not have supportive family structures in place. • Help-seeking behaviour should be targeted; for example, through the provision of accurate information (e.g. during antenatal classes) and by improving public awareness of PP.

### Strengths and limitations

The use of thematic synthesis for this review enhanced the rigour and transparency of the analysis. It allowed for the incorporation of multiple findings and the subsequent offer of a new interpretation which can be used to inform improvements in clinical practice. The systematic search was comprehensive, minimizing language and publication biases. All studies included were of high methodological quality, thereby enhancing the trustworthiness of the data synthesised. Furthermore, steps were taken to maximize transparency and enhance rigour, including the use of data management software and independent reviewers (Tong et al. 2012).

The findings report on the experiences of 103 women and 42 family members and capture a broad range of experiences up to 32 years postpartum. This is viewed as a strength of the review, because it allowed for the analysis and interpretation of a diverse range of views and experiences. However, it was not possible to investigate individuals’ experiences specifically in relation to their stage of recovery. It is therefore unclear how an individual’s perspective may change over time.

Despite not limiting studies by language, only research from the UK, USA, Canada and Sweden, was identified. This means that the experiences of women and family living in other countries, including less developed countries, perhaps with different family structures and healthcare systems are unknown. Caution is therefore advised when generalizing findings. Despite applying no limits on date, no research pre-dated 2003. This may reflect the poor representation of qualitative research in medical literature (Shuval et al. 2011) which is now on the increase due to an increased recognition on its value (Popay 2006). Furthermore, the analysis is limited to the information provided in the studies included in this review. Other topics which may be pertinent, for example, regarding the types of services accessed, were often not clearly stated.

### Further research

This review highlights a number of gaps in the literature in which further research is needed. Research should focus on the types of long-term psychological and psychosocial interventions that women may find helpful during their recovery from an episode of PP. Exploration may be required to further understand the professional perspective and how this can be integrated into the conceptual model. Family support could be investigated due to their influential role, with emphasis on how to support and enhance their position. It may also be helpful to investigate the experiences of women with different types of family and healthcare support, and to explore the perceived barriers and facilitators to accessing professional input. Finally, it may be useful to explore the impact of preconception counselling and to investigate the factors involved in accessing this type of support.

## Conclusions

The review reveals the complexity of recovery from PP and the need for intervention to be incorporated into a longer term recovery plan, which includes psychological and psychosocial needs alongside medical management. The results highlight the core need for women to assimilate and adjust to their new role as a mother, whilst integrating their experience of PP and associated sense of loss, which could be facilitated through psychological input. The review further depicts a central role for family and reveals an overlap in their reported experiences, suggesting that more input should be provided in collaboration with the family, with the aim to enhance their relationships and ways of coping. The findings also highlight the importance of healthcare professionals providing a timely assessment and providing the necessary support which incorporates the needs of the woman, baby and family.
